# Effect of maternal age, embryo number and quality on pregnancy outcome during frozen embryo transfer cycle

**DOI:** 10.3389/fendo.2025.1596178

**Published:** 2025-11-07

**Authors:** Wenyi Gao, Weiwei Tang, Caixia Li, Yun Deng, Yao Chen, Yanru Zhang, Xiaohong Pan, Chunyan Yang, Yuanyuan Liu, Fang Xiong, Xin Jin

**Affiliations:** 1 The 904th Hospital of the Joint Logistic Support Force of the People's Liberation Army (PLA), Reproductive Center, Wuxi, China; 2 Wuxi Maternity and Child Health Care Hospital, Reproductive Center, Wuxi, China

**Keywords:** maternal age, embryo number, embryo quality, clinical pregnancy rate, implantation rate, live birth rate

## Abstract

**Background:**

To investigate the effects of maternal age, the number of transferred embryos, and embryo quality on pregnancy-related outcomes in frozen-thawed embryo transfer cycles.

**Design:**

A retrospective cohort study was conducted on 1031 frozen-thawed embryo transfer cycles enrolled at our hospital from January 2015 to December 2021.

**Results:**

In the pregnant group, maternal age was significantly lower compared to the non-pregnant group; additionally, both the number of transferred embryos and the number of high-quality embryos transferred were significantly higher in the pregnant group. Women older than 35 years exhibited significantly lower clinical pregnancy rate (p < 0.05), implantation rate (p < 0.001), and live birth rate (p < 0.01). A higher number of transferred embryos was associated with significantly increased clinical pregnancy rate (p < 0.001) and live birth rates (p < 0.001). When transferring two embryos, higher numbers of 7–9 cell embryos (p < 0.01) and grade 1 embryos (p < 0.001) were positively correlated with improved clinical outcomes, while increased transfers of fragmented (p < 0.05) and uneven embryos (p < 0.05) were negatively associated with these outcomes.

**Conclusion:**

Maternal age, the number of transferred embryos, and embryo quality significantly influence pregnancy outcomes in frozen-thawed embryo transfer cycles. Clinicians should carefully tailor transfer plans based on individual patient characteristics and select optimal embryos for transfer to maximize success rate.

## Introduction

The ultimate goal of assisted reproductive technology (ART) is to achieve a full-term, healthy singleton pregnancy (1). With the continuous advancement of ART, frozen-thawed embryo transfer (FET) has emerged as a mainstream approach in clinical practice (2). However, determining optimal transfer strategies and selecting the most suitable frozen-thawed embryos to maximize pregnancy outcomes remain significant challenges and have become a focal point of research interest among scholars. While previous studies have explored various factors influencing FET success, such as embryo quality and quantity (3–5), there remains a paucity of comprehensive research examining the interplay between maternal age, embryo characteristics, and pregnancy outcomes in FET cycles. Additionally, existing studies often overlook the nuanced physiological differences between younger and older women, which can significantly impact embryo implantation and pregnancy success.

This study aims to investigate the effects of maternal age, embryo number, and quality on pregnancy outcomes in FET cycles. By addressing these gaps in the literature, our research seeks to provide valuable insights for clinicians in developing personalized embryo selection and transfer protocols tailored to individual patients’ profiles. The findings of this study will contribute to optimizing FET strategies and improving clinical outcomes, ultimately enhancing the chances of achieving a successful singleton pregnancy for couples undergoing ART.

## Materials and methods

### Research object

A retrospective analysis was performed on 1031 cycles of freeze-thaw embryo transfer to facilitate pregnancy in the 904th Hospital of the Joint Logistic Support Force of the Chinese People’s Liberation Army from January 2015 to December 2021.

The inclusion criteria encompassed patients who underwent complete superovulation, oocyte retrieval, IVF treatment, and freeze-thaw embryo transfer procedures at the 904th Hospital of the Joint Logistic Support Force of the Chinese People’s Liberation Army, with fully documented clinical records; these patients were diagnosed with infertility attributable to tubal factors, unexplained etiologies, or male factors, and had no prior history of adverse pregnancy outcomes (e.g., ectopic pregnancy, embryonic arrest) or recurrent habitual abortions (defined as ≥2 clinically confirmed spontaneous pregnancy losses). While patients were excluded if they had a history of ovarian surgery or tumor, were identified as egg donors, presented with active infectious diseases (e.g., HIV, hepatitis B/C), exhibited uterine cavity adhesions (confirmed via hysteroscopy or sonohysterography), demonstrated ovarian function decline (AMH <1.1 ng/mL) or polycystic ovary syndrome (Rotterdam criteria), or were diagnosed with hydrosalpinx (unilateral or bilateral tubal fluid accumulation ≥10 mm on transvaginal ultrasound).

The study was approved by ethical requirements of the 904th Hospital of the Joint Logistic Support Force of the Chinese People’s Liberation Army, and all patients and their families have given informed consent.

### Treatment of freeze-thaw embryo transfer

Based on the transfer strategy provided by the clinical team, the cleavage-stage embryos selected for transfer are thawed using the vitrification thawing method. After thawing, the embryos are observed under a microscope to assess their quality prior to transfer. The grading for cleavage-stage embryos was performed using the Society for Assisted Reproductive Technology (SART) scoring method (6, 7), according to which grade I and II embryos were classified as high-quality.

12–14 days after transplantation, blood samples are taken to test for human chorionic gonadotropin (hCG). If the test is positive, an ultrasound examination is performed 4–6 weeks later. If a gestational sac is visible, it indicates a clinical pregnancy. Follow-up is conducted until 12 months after the transfer.

### Grouping method and observation indicators

Based on the occurrence of clinical pregnancy, 1031 treatment cycles were categorized into a pregnancy group and a non-pregnancy group. The general indicators, as well as the number of embryo transfers and high-quality embryos, were compared between the two groups. Grouping was also performed based on the number of embryo transfers and high-quality embryos, and the general indicators and clinical pregnancy indicators were compared among the groups. Furthermore, grouping was also performed based on embryo quality, and a comparison of clinical pregnancy indices was undertaken among the respective groups.

The general indices encompassed the age of the female patient, female patient’s body mass index (BMI), the duration of infertility, and the endometrial thickness on the day of transfer. Clinical pregnancy indices, on the other hand, included the clinical pregnancy rate(CPR), embryo implantation rate (IR), live birth rate (LBR), biochemical pregnancy rate, and miscarriage rate. The calculation methods for these indicators adhered to the expert consensus on quality control of key indices in embryo laboratories (6).

### Statistical methods

All quantitative data were expressed as mean ± SD, and comparisons between two groups were performed using the Student’s t-test, while comparisons among multiple groups were conducted using one-way ANOVA. Count data were presented as percentages, and comparisons between two groups as well as among multiple groups were both conducted using the χ2 test. All statistical analyses were carried out using GraphPad Prism software and SPSS 29.0 software, with graphs generated using GraphPad Prism software. A P-value ≥0.05 was considered statistically non-significant, whereas a P-value <0.05 was considered statistically significant.

## Results

### Comparison between pregnant group and non-pregnant group

A total of 1,031 freeze-thaw embryo transfer cycles were analyzed, with 469 cycles (45.5%) achieving clinical pregnancy and 562 cycles (54.5%) not reaching clinical pregnancy. Comparative analysis of baseline characteristics revealed that maternal age was significantly lower in the clinical pregnancy group compared to the non-clinical pregnancy group (t=2.312, P<0.05; **Table 1**). No other significant differences were observed between the groups in terms of female BMI, duration of infertility, or endometrial thickness (all P>0.05; **Table 1**).

**Table 1:** Comparison of the basic information per cycle between pregnant and non-pregnant groups.

Group	Pregnant	Non-pregnant	*t*	*P*
N	469	562		
maternal age (years)	30.89 ± 0.22^a^	31.61 ± 0.22^b^	2.31	0.021
Female BMI(kg/m^2^)	22.82 ± 0.17^a^	22.80 ± 0.14^a^	0.11	0.91
Duration of infertility(years)	4.23 ± 0.14^a^	4.45 ± 0.14^a^	1.13	0.26
Endometrial thickness(mm)	9.47 ± 0.07^a^	9.38 ± 0.06^a^	0.97	0.33

BMI, female body mass index.

The difference of basic parameters among groups with different superscript letters was statistically significant (P < 0.05), while the difference of basic parameters among groups with the same superscript letters was not statistically significant (P > 0.05).

The number of embryos transferred per cycle in the pregnancy group was significantly higher than that in the non-pregnancy group (t = 4.092, P < 0.0001). Significant differences were also found in the distribution of transferred embryo numbers between the two groups (χ² = 17.874, P < 0.001; **Table 2**).

**Table 2:** Comparison of the number of embryos transferred per cycle between pregnant and non-pregnant groups.

Number of embryos transferred		Group		*t*/*χ2*	*P*
		Pregnant	Non-pregnant		
	average	2.12 ± 0.02	2.00 ± 0.02	4.09	< 0.0001
	1	21(4.48)^a^	59(10.50)^b^	17.87	<0.001
	2	372(79.32)^a^	443(78.83)^a^		
	3	76(16.20)^a^	60(10.68)^b^		

The difference of basic parameters among groups with different superscript letters was statistically significant (P < 0.05), while the difference of basic parameters among groups with the same superscript letters was not statistically significant (P > 0.05).

The number of high-quality embryos transferred per cycle in the pregnancy group was significantly higher than that in the non-pregnancy group (t = 5.753, P < 0.0001). Significant differences were also found in the distribution of transferred high-quality embryos between the two groups (χ² = 34.781, P < 0.001; **Table 3**). Specifically, the proportions of transferring zero or one high-quality embryo were significantly lower in the pregnancy group compared to the non-pregnancy group, while the proportions of transferring two or three high-quality embryos were significantly higher in the pregnancy group.

**Table 3:** Number of high-quality embryos transferred in pregnancy group and non-pregnancy group.

Number of high-quality embryos		Group		*t*/*χ2*	*P*
		Pregnant	Non-pregnant		
	average	1.90 ± 0.03	1.66 ± 0.03	5.75	< 0.0001
	0	22(4.69)^a^	52(9.25)^b^	34.781	<0.001
	1	57(12.15)^a^	123(21.89)^b^		
	2	334(71.22)^a^	354(62.99)^b^		
	3	56(11.94)^a^	33(5.87)^b^		

The difference of basic parameters among groups with different superscript letters was statistically significant (P < 0.05), while the difference of basic parameters among groups with the same superscript letters was not statistically significant (P > 0.05).

### Influence of maternal age on pregnancy outcome

Cases were categorized into three groups based on maternal age. Group 1 (n = 402) consisted of women aged <30 years, Group 2 (n = 366) included women aged 30–34 years, and Group 3 (n = 263) comprised women aged ≥35 years. Significant differences were observed among the three groups in CPR, IR, and LBR (P < 0.001; **Table 4**). Specifically:

**Table 4:** Influence of maternal age on pregnancy outcome.

Group	1	2	3	*t*	*P*
Number of cases	402	366	263		
Transferred embryos number	824	752	542		
CPR	202(50.2)^a^	164(44.8)^ab^	103(39.2)^b^	7.99	0.018
IR	274(33.3)^a^	211(28.1)^b^	123(22.7)^c^	18.05	<0.001
LBR	178(44.3)^a^	138(37.7)^a^	78(29.7)^b^	14.46	0.001
Biochemical pregnancy rate	12(3.0)^a^	11(3.00)^a^	6(2.30)^a^	0.37	0.83
Miscarriage rate	24(6.0)^a^	26(7.1)^a^	24(9.1)^a^	2.38	0.30

CPR, clinical pregnancy rate; IR, embryo implantation rate; LBR, live birth rate.

The difference of basic parameters among groups with different superscript letters was statistically significant (P < 0.05), while the difference of basic parameters among groups with the same superscript letters was not statistically significant (P > 0.05).

- The CPR in Group 3 was significantly lower than that in Group 1.
- The IR in Group 3 was significantly lower than that in Group 2, which was itself significantly lower than that in Group 1.
- The LBR in Group 3 was significantly lower than both Group 1 and Group 2.

No significant differences were found among the three groups in biochemical pregnancy rate and miscarriage rate (P > 0.05; **Table 4**). Although the miscarriage rate showed a trend of increasing with advancing age (Group 3 > Group 2 > Group 1), these differences did not reach statistical significance.

### Impact of the number of embryos transferred on pregnancy outcomes

Cases were categorized into three groups based on the number of embryos transferred. Group 1 (n = 80) consisted of cases with a single embryo transferred, Group 2 (n = 815) included cases with double embryos transferred, and Group 3 (n = 136) comprised cases with triple embryos transferred. Baseline characteristics such as maternal age, female BMI, duration of infertility, and endometrial thickness were comparable across the three groups (P > 0.05; **Table 5**).

**Table 5:** Influence of transferred embryos numbers on pregnancy outcome.

Transferred embryos numbers	1	2	3	χ2	*P*
Number of cases	80	815	136		
maternal age (years)	32.10 ± 0.67^a^	31.12 ± 0.17^a^	31.79 ± 0.42^a^	2.24	0.11
Female BMI(kg/m^2^)	22.43 ± 0.44^a^	22.77 ± 0.13^a^	22.92 ± 0.32^a^	0.46	0.63
Duration of infertility(years)	4.94 ± 0.40^a^	4.26 ± 0.11^a^	4.54 ± 0.26^a^	1.92	0.15
Endometrial thickness(mm)	9.54 ± 0.19^a^	9.40 ± 0.05^a^	9.45 ± 0.12^a^	0.37	0.69
Transferred embryos number	80	1630	408		
CPR	21(26.3)^a^	372(45.6)^b^	76(55.9)^c^	17.87	<0.001
IR	21(26.3)^a^	477(29.3)^a^	110(27.0)^a^	1.09	0.58
LBR	15(18.8)^a^	313(38.4)^b^	66(48.5)^c^	18.98	<0.001
Biochemical pregnancy rate	1(1.25)^a^	24(2.94)^a^	4(2.94)^a^	0.78	0.68
Miscarriage rate	6(7.50)^a^	59(7.24)^a^	9(6.62)^a^	0.08	0.96

BMI, female body mass index; CPR, clinical pregnancy rate; IR, embryo implantation rate; LBR, live birth rate.

The difference of basic parameters among groups with different superscript letters was statistically significant (P < 0.05), while the difference of basic parameters among groups with the same superscript letters was not statistically significant (P > 0.05).

Significant differences were observed in CPR and LBR among the three groups (P < 0.001; **Table 5**). Both CPR and LBR increased significantly with the increase in the number of transferred embryos. However, no significant differences were found in IR, biochemical pregnancy rate, and abortion rate among the three groups (P > 0.05; **Table 5**). Notably, the IR in Group 2 (double embryos transferred) was slightly higher than that in Groups 1 and 3, but this difference did not reach statistical significance.

### Impact of the number of high-quality embryos transferred on pregnancy outcomes

Cases were divided into four groups based on the number of high-quality embryos transferred. Group 1 (n = 74) consisted of cases with no high-quality embryo transferred, Group 2 (n = 180) included cases with one high-quality embryo transferred, Group 3 (n = 688) comprised cases with two high-quality embryos transferred, and Group 4 (n = 89) included cases with three high-quality embryos transferred. Baseline characteristics such as maternal age, female BMI, duration of infertility, and endometrial thickness were comparable across the four groups (P > 0.05; **Table 6**). Significant differences were observed in CPR, IR, and LBR among the four groups (P < 0.001; **Table 6**). However, no significant differences were found in biochemical pregnancy rate and miscarriage rate among the four groups (P > 0.05; **Table 6**).

**Table 6:** Influence of transferred high-quality embryos numbers on pregnancy outcome.

Variable	Number of high-quality embryos				χ2	*P*
	0 (n=74)	1(n=180)	2(n=688)	3(n=89)		
maternal age (years)	31.68 ± 0.63^a^	32.03 ± 0.39^a^	31.00 ± 0.18^a^	31.64 ± 0.56^a^	2.45	0.06
Female BMI(kg/m^2^)	22.78 ± 0.49^a^	22.99 ± 0.25^a^	22.70 ± 0.14^a^	22.79 ± 0.40^a^	0.31	0.82
Duration of infertility(years)	5.25 ± 0.38^a^	4.50 ± 0.25^ab^	4.22 ± 0.12^b^	4.30 ± 0.33^ab^	2.44	0.06
Endometrial thickness(mm)	9.44 ± 0.23^a^	9.51 ± 0.11^a^	9.39 ± 0.05^a^	9.39 ± 0.16^a^	0.31	0.82
Transferred embryos number	138	311	1402	267		
CPR	22(29.7)^a^	57(31.7)^a^	334(48.5)^b^	56(62.9)^c^	34.78	<0.001
IR	26(18.8)^a^	62(19.9)^a^	436(31.1)^b^	84(31.5)^b^	23.16	<0.001
LBR	18(24.3)^a^	43(23.9)^a^	283(41.1)^b^	50(56.2)^c^	36.34	<0.001
Biochemical pregnancy rate	0(0.00)^a^	3(1.67)^a^	25(3.63)^a^	1(1.12)^a^	5.63	0.13
Miscarriage rate	4(5.41)^a^	14(7.78)^a^	50(7.27)^a^	6(6.74)^a^	0.48	0.92

BMI, female body mass index; CPR, clinical pregnancy rate; IR, embryo implantation rate; LBR, live birth rate.

The difference of basic parameters among groups with different superscript letters was statistically significant (P < 0.05), while the difference of basic parameters among groups with the same superscript letters was not statistically significant (P > 0.05).

### Receiver operating characteristic curve analysis of maternal age, embryo number and quality for predicting pregnancy outcomes

**Figure 1:**
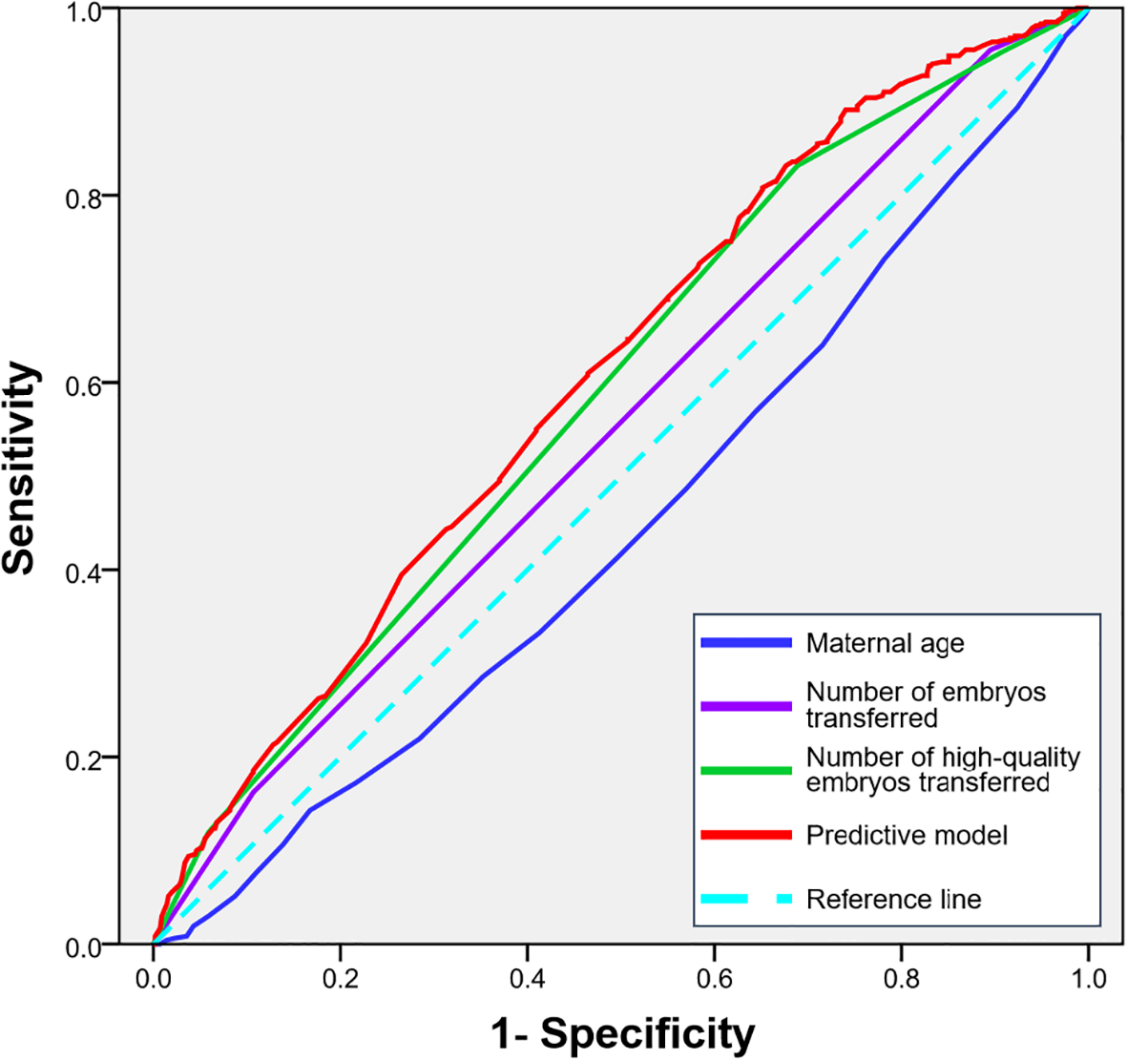
ROC curves for predicting clinical pregnancy based on maternal age, the number of embryos transferred and the number of high-quality embryos transferred.

To quantify the predictive power of maternal age, embryo number and quality on pregnancy outcomes and to identify potential clinical thresholds, we performed Receiver Operating Characteristic (ROC) curve analysis. The analysis for female age yielded an area under the curve (AUC) of 0.446 (95% CI, 0.411-0.481; P = 0.003), indicating limited predictive ability. The predictive strength of the number of embryos transferred alone showed an AUC of 0.552 (95% CI, 0.517-0.587; P = 0.004). Notably, the number of high-quality embryos transferred demonstrated a superior predictive capacity compared to the total number alone, with an AUC of 0.589 (95% CI, 0.554-0.632; P < 0.001). A combined predictive model integrating female age and the number of high-quality embryos achieved the highest performance, with an AUC of 0.607 (95% CI, 0.574-0.641; P < 0.001). The ROC curves for all parameters are presented in **Figure 1**.

Embryonic morphology is a critical criterion for assessing embryo quality, encompassing several key indicators: cell number, embryo grade, symmetry, and fragmentation. Each of these factors plays a significant role in determining the developmental potential of an embryo. The number of cells in an embryo at a specific developmental stage is a key indicator of its quality. For example, at Day 3 of development, a high-quality embryo typically consists of 7–9 cells. This range is considered optimal for ensuring proper development and implantation potential. The even distribution of cells and overall symmetry of the embryo are strong indicators of its health. Symmetrical embryos are generally associated with better developmental outcomes, as they reflect balanced cellular growth and differentiation. Fragmentation refers to the presence of cellular debris or dead cells within the embryo. Minimal fragmentation is highly favorable, as excessive fragmentation can impair embryo viability and reduce the chances of successful implantation.

### Impact of embryo cell number on pregnancy outcomes

In this retrospective analysis of 815 embryo transfer cycles, cases were stratified based on the number of Day 3 7–9 cell embryos transferred. The cohort comprised 140 cycles (17.2%) without any 7–9 cell embryos (Group 0), 179 cycles (22.0%) with one 7–9 cell embryo (Group 1), and 496 cycles (60.8%) with two 7–9 cell embryos (Group 2). Notably, we observed significant intergroup variations in key clinical outcomes. Both CPR (P<0.01) and IR (P<0.001) demonstrated progressive improvement with increasing number of 7–9 cell embryos transferred. LBR followed a similar pattern, showing statistically significant differences among groups (P<0.01) (**Table 7**). However, biochemical pregnancy rate (P = 0.32) and miscarriage rate (P = 0.47) remained comparable across all three groups (**Table 7**).

**Table 7:** Influence of transferred 7–9 cell embryos numbers on pregnancy outcome.

7–9 cell embryos number	0	1	2	χ2	*P*
Number of cases	140	179	496		
Transferred embryos number	280	358	992		
CPR	51(36.4)^a^	74(41.3)^ab^	247(49.8)^b^	9.58	0.008
IR	59(21.1)^a^	87(24.3)^a^	331(33.4)^b^	21.41	<0.001
LBR	39(27.9)^a^	63(35.2)^ab^	211(42.5)^b^	10.95	0.004
Biochemical pregnancy rate	4(2.86)^a^	5(2.79)^a^	15(3.02)^a^	0.029	0.99
Miscarriage rate	12(8.57)^a^	11(6.15)^a^	36(7.26)^a^	0.69	0.71

CPR, clinical pregnancy rate; IR, embryo implantation rate; LBR, live birth rate.

The difference of basic parameters among groups with different superscript letters was statistically significant (P < 0.05), while the difference of basic parameters among groups with the same superscript letters was not statistically significant (P > 0.05).

### Impact of embryo grade on pregnancy outcomes

Cases were stratified based on the number of Grade 1 embryos transferred (total cycles=815). The cohort comprised 290 cycles (35.6%) without any Grade 1 embryos (Group 0), 167 cycles (20.5%) with one Grade 1 embryo (Group 1), and 358 cycles (43.9%) with two Grade 1 embryos (Group 2). Notably, we observed significant intergroup variations in key clinical outcomes. Both CPR (P<0.001), IR (P<0.001) and LBR (P<0.001) demonstrated progressive improvement with increasing numbers of Grade 1 embryos transferred (**Table 8**). However, biochemical pregnancy rate (P = 0.84) and miscarriage rate (P = 0.91) remained comparable across all three groups (**Table 8**).

**Table 8:** Influence of transferred grade 1 embryos numbers on pregnancy outcome.

Grade 1 embryos number	0	1	2	χ2	*P*
Number of cases	290	167	358		
Transferred embryos number	580	334	716		
CPR	91(31.4)^a^	79(47.3)^b^	202(56.4)^b^	40.74	<0.001
IR	112(19.3)^a^	97(29.0)^b^	268(37.4)^c^	50.83	<0.001
LBR	77(26.6)^a^	64(38.3)^b^	172(48.0)^c^	31.29	<0.001
Biochemical pregnancy rate	6(2.07)^a^	6(3.59)^a^	12(3.35)^a^	1.23	0.54
Miscarriage rate	14(4.83)^a^	15(8.98)^ab^	39(10.89)^b^	3.96	0.14

CPR, clinical pregnancy rate; IR, embryo implantation rate; LBR, live birth rate.

The difference of basic parameters among groups with different superscript letters was statistically significant (P < 0.05), while the difference of basic parameters among groups with the same superscript letters was not statistically significant (P > 0.05).

### Impact of embryo fragmentation on pregnancy outcomes

In this retrospective analysis of 815 embryo transfer cycles, cases were stratified based on the number of fragmented embryos transferred. The cohort comprised 609 cycles (74.7%) without any fragmented embryos (Group 0), 121 cycles (14.8%) with one fragmented embryo (Group 1), and 85 cycles (10.4%) with two fragmented embryos (Group 2). Notably, we observed progressive decline in clinical outcomes. Both CPR (P<0.05), IR (P<0.05) and LBR (P<0.05) demonstrated dose-dependent reduction with increasing numbers of Grade 1 embryos transferred (**Table 9**). However, biochemical pregnancy rate (P = 0.92) and miscarriage rate (P = 0.49) remained comparable across all three groups (**Table 9**).

**Table 9:** Influence of fragmented embryos numbers on pregnancy outcome.

Fragmented embryos number	0	1	2	χ2	*P*
Number of cases	609	121	85		
Transferred embryos number	1218	242	170		
CPR	294(48.3)^a^	49(40.5)^ab^	29(34.12)^b^	7.54	0.02
IR	378(31.0)^a^	59(24.4)^b^	40(23.5)^b^	7.33	0.03
LBR	250(41.1)^a^	38(31.4)^b^	25(29.4)^b^	7.22	0.03
Biochemical pregnancy rate	18(2.96)^a^	4(3.31)^a^	2(2.35)^a^	0.16	0.92
Miscarriage rate	44(7.22)^a^	11(9.09)^a^	4(4.71)^a^	1.43	0.49

CPR, clinical pregnancy rate; IR, embryo implantation rate; LBR, live birth rate.

The difference of basic parameters among groups with different superscript letters was statistically significant (P < 0.05), while the difference of basic parameters among groups with the same superscript letters was not statistically significant (P > 0.05).

### Impact of embryo uniformity on pregnancy outcomes

This study analyzed 815 embryo transfer cycles classified according to the number of unevenly developed embryos transferred. The study population consisted of 610 cycles (74.8%) without any uneven embryos (Group 0), 155 cycles (19.0%) with one uneven embryo (Group 1), and 50 cycles (6.1%) containing two uneven embryos (Group 2). Importantly, clinical parameters revealed progressive deterioration with increasing developmental irregularity: CPR (P<0.05), IR (P<0.05) and LBR (P<0.05) all declined. Biochemical pregnancy rate (P = 0.62) and miscarriage rate (P = 0.74) showed comparable rates across all cohorts (**Table 10**).

**Table 10:** Influence of uneven embryos numbers on pregnancy outcome.

Uneven embryos number	0	1	2	χ2	*P*
Number of cases	610	155	50		
Transferred embryos number	1220	310	100		
CPR	292(47.9)^a^	64(41.3)^ab^	16(32.0)^b^	6.153	0.046
IR	379(31.1)^a^	80(25.8)^ab^	18(18.0)^b^	9.833	0.007
LBR	249(40.8)^a^	53(34.2)^ab^	11(22.0)^b^	8.354	0.015
Biochemical pregnancy rate	20(3.28)	3(1.94)	1(2.00)	0.947	0.62
Miscarriage rate	43(7.05)	11(7.10)	5(10.00)	0.605	0.74

CPR, clinical pregnancy rate; IR, embryo implantation rate; LBR, live birth rate.

The difference of basic parameters among groups with different superscript letters was statistically significant (P < 0.05), while the difference of basic parameters among groups with the same superscript letters was not statistically significant (P > 0.05).

### ROC curve analysis of specific embryo morphological parameters for predicting pregnancy outcomes

To determine the relative importance of specific embryo morphological features, we performed ROC curve analysis for the number of 7–9 cell embryos, Grade 1 embryos, fragmented embryos, and uneven embryos transferred.

The number of Grade 1 embryos transferred demonstrated the strongest predictive power for clinical pregnancy among all morphological parameters, with an AUC of [0.619 (95% CI, 0.580-0.657; P<0.01). The number of 7–9 cell embryos also showed significant predictive value, with an AUC of [0.555] (95% CI, 0.515-0.594; P = 0.007).In contrast, the parameters indicating suboptimal development—namely, the number of fragmented embryos (AUC: 0.464; 95% CI, 0.425-0.545; P = 0.078) and uneven embryos (AUC: 0.458; 95% CI, 0.419-0.498; P = 0.040)—showed considerably lower discriminatory capacity.

**Figure 2:**
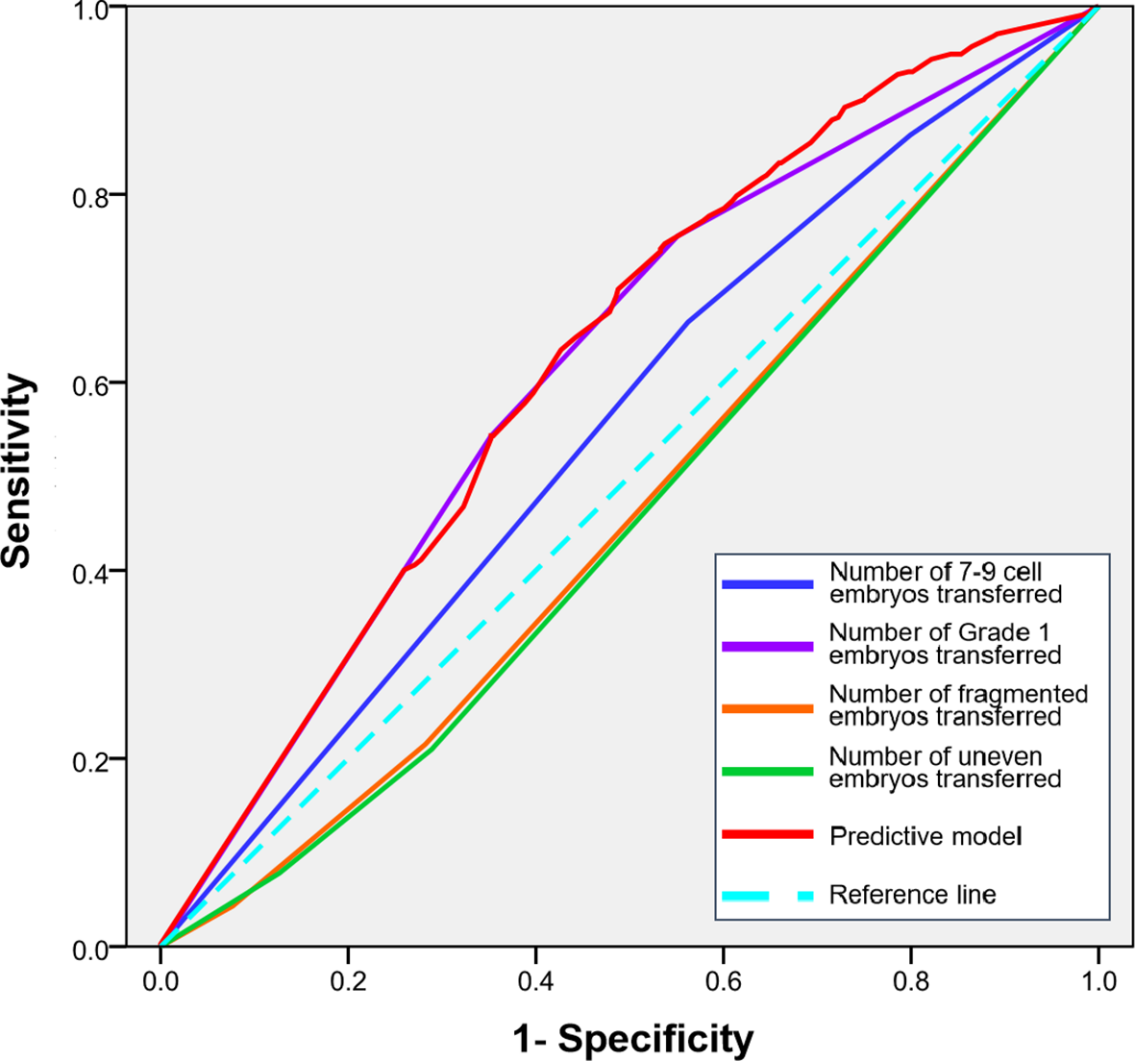
ROC curves for predicting clinical pregnancy based on embryo morphological parameters.

A combined predictive model integrating these specific embryo morphological features achieved the highest performance, with an AUC of 0.628 (95% CI, 0.590-0.666; P < 0.001). The ROC curves illustrating the predictive performance of these individual morphological criteria are presented in **Figure 2**.

## Discussion

### Impact of maternal age on pregnancy outcomes following frozen-thawed embryo transfer

In assisted reproductive technology (ART), maternal age has always been a crucial factor closely monitored by clinicians due to its significant influence on treatment outcomes. A multivariate logistic analysis identified a low CPR in patients older than 35 years (8). Similarly, Zacà C (9) demonstrated that there were statistically significant differences in total good blastocyst development rate (TGBDR) and cumulative CPR among different age groups. The significance of maternal age is evident not only in *in vitro* fertilization (IVF) and fresh embryo transfer cycles but also in frozen-thawed embryo transfer (FET) cycles. According to Holschbach V (10), in FET cycles, maternal age is an independent predictive factors of frozen embryo transfer outcome. In 2022, a study showed that maternal age independently associated with pregnancy outcomes, even in cycles that transferred a top-grade embryo (11). This finding aligns with the results of our study, which indicate a marked decline in CPR, IR, and LBR among women aged over 35 years, underscoring the importance of studying pregnancy outcomes from multiple perspectives. Furthermore, our study compared patient ages between the pregnant and non-pregnant groups and found that women in the pregnant group were significantly younger than those in the non-pregnant group, providing bidirectional evidence for the impact of maternal age on pregnancy outcomes in FET cycles.

However, there are also dissenting views. Irani M (12) argued that maternal age affects the number of euploid embryos, but does not affect their implantation potential. They attributed this with the object including 785 patients who underwent frozeno-thawed euploid embryo transfer, which is difference with our study.

### The impact of the transferred embryos number on pregnancy outcomes

The influence of the embryos number on pregnancy outcomes is foreseeable, and some scholars have previously conducted research to demonstrate this. Earlier studies have shown that, regardless of whether it is fresh embryo transfer or frozen-thawed embryo transfer, the CPR for double embryo transfer is significantly higher compared to single embryo transfer (13). In 2021, Namath A (14) also published a paper indicating that compared to transferring a single frozen-thawed embryo, transferring two frozen-thawed embryos led to a significant increase in LBR, but with a corresponding increase in preterm birth rates and lower birth weights.

This study jointly analyzed the number of embryos transferred and the number of high-quality embryos transferred. With an increase in the number of embryos transferred, while factors such as maternal age, BMI, duration of infertility, and endometrial thickness remained unchanged, both CPR and LBR increased accordingly. Similarly, as the number of high-quality embryos transferred increased, with the aforementioned factors remaining constant, CPR, IR, and LBR also increased. Additionally, this study compared the number of embryos and high-quality embryos transferred between the pregnant group and the non-pregnant group. The pregnant group had a higher number of embryos and high-quality embryos transferred compared to the non-pregnant group. Specifically, the number of cases with 0~1 high-quality embryos transferred in the pregnant group was significantly lower than that in the non-pregnant group, while the number of cases with 2 ~3 high-quality embryos transferred in the pregnant group was significantly higher. In this way, the study verified the impact of the number of embryos transferred and the number of high-quality embryos transferred on pregnancy outcomes during frozen embryo transfer cycles from both positive and negative perspectives.

The findings of this study are similar to those of Zhu Q (4), which showed that the LBR was higher when transferring two high-quality embryos compared to one high-quality and one non-high-quality embryo. Furthermore, transferring two embryos (one high-quality and one non-high-quality) resulted in a higher LBR than transferring a single high-quality embryo, and transferring a single high-quality embryo led to a higher LBR than transferring a single non-high-quality embryo. However, guidelines for the number of embryos to transfer following *in vitro* fertilization state that in order to minimize the occurrence of multiple pregnancies, the number of embryos transferred should be determined according to the specific circumstances, and may be appropriately increased in patients with advanced age or poor prognosis (15).

Although our research results demonstrate a positive correlation between the number of embryos transferred and clinical pregnancy rates, this strategy must be contextualized within the critical framework of the risk of multiple pregnancies. The increase in pregnancy success rates associated with the transfer of more than one embryo inevitably elevates the possibility of twin and higher-order multiple pregnancies. This is a significant concern, as multiple pregnancies are unequivocally associated with elevated maternal risks (such as preeclampsia, gestational diabetes, and preterm labor) and neonatal complications including prematurity (<37 weeks), low birth weight (<2,500 grams), and long-term developmental issues (16). Consequently, the pursuit of a higher pregnancy rate must be carefully balanced against the imperative to minimize iatrogenic complications. Our results, which confirm the efficacy of transferring multiple embryos, thus underscore the crucial importance of continuously optimizing the embryo selection criteria. The recommendations from the American Society for Reproductive Medicine and SART on limiting the number of embryos transferred have been revised several times to increase the use of elective Single Embryo Transfer (eSET) (17). Future research should focus on refining predictive models to identify patients who can maintain a high success rate and almost eliminate the risk of multiple pregnancies by undergoing eSET.

### Impact of transferred embryo quality on pregnancy outcomes

This study delves into the influence of embryo quality on pregnancy outcomes, examining four key aspects: embryo grade, number of blastomeres, fragmentation, and cleavage symmetry. Regarding the number of blastomeres and the clinical outcomes of frozen-thawed embryo transfer, there exists a diversity of opinions. The normal developmental trajectory for human embryos involves reaching the 7–9 cell stage by the third day post-fertilization (18, 19). Zhu XL (20) demonstrated in their 2021 paper that the CPR for embryos with 8 or more cells transferred on the third day was significantly higher than those with fewer than 8 cells. Other studies have also shown that increased blastomere number on the third day was associated with higher LBR (21). This study found that the group receiving two 7–9 cell embryos had significantly higher CPR, IR, and LBR than other groups. This may be attributed to the degree of synchronization between embryo development and endometrial receptivity (22, 23).

Previous research has underscored the significance of fragmentation in transplantation pregnancy outcomes. Fragmented embryos exhibit significantly lower clinical pregnancy and implantation rates compared to non-fragmented embryos (24). Scholars have discovered that removing fragments from fragmented embryos two hours prior to transfer leads to an increase in CPR, with no statistically significant difference compared to transferring non-fragmented embryos (25).Aldemir O (26) suggested that the rate of embryo fragmentation correlated with IL-6 levels in follicular fluid, which could lead to a significant reduction in CPR. This study’s results indicate that the group receiving two fragmented embryos had significantly lower CPR, IR, and LBR compared to other groups. Fragmentation is correlated with embryonic developmental potential, with an increase in nuclear fragments leading to a gradual decline in this potential. Embryos with over 25% fragmentation rarely form blastocysts (27). Additionally, as fragmentation increases, so does the rate of chromosomal abnormalities (28).

There are limited reports on the impact of cleavage symmetry on pregnancy outcomes. Only studies on early cleavage suggest that asymmetric cleavage leads to reduced clinical pregnancy and implantation rates, making it an effective and valuable method for predicting clinical pregnancy outcomes (29). Liu J (30) also believe that unequal-sized blastomeres on the third day of cleavage can impair embryonic development and reduce pregnancy rates, through a retrospective study. This study found that the group receiving two symmetric embryos had significantly higher CPR, IR, and LBR than other groups. This may be due to asymmetric cleavage potentially leading to a significant increase in embryonic aneuploidy rates (31).

The embryo grade is evaluated according to the Gardner method, which is directly related to factors such as the number of blastomeres, fragmentation, and uniformity (6, 7). This study found that the CPR, IR, and LBR in the group with two grade 1 embryos transferred were significantly higher than those in other groups. Its impact on pregnancy outcomes is closely related to these three factors.

In summary, maternal age, the number of embryos transferred, and the quality of embryos transferred are all important factors affecting the pregnancy outcomes of frozen-thawed embryo transfer. Among them, the quality of embryos transferred includes embryo grade, number of blastomeres, fragmentation, and asymmetric cleavage. In clinical practice, a multi-angle approach should be taken to carefully formulate embryo transfer strategies and select appropriate embryos.

## Conclusion

The present study explored the effects of maternal age, embryo quantity and embryo quality on pregnancy outcome during freeze-thaw embryo transfer cycle. First of all, maternal age is a major factor affecting the CPR, IR, and LBR, especially when the maternal age is over 35 years old, the pregnancy outcomes of freeze-thaw embryo transfer cycle is worse than that of young women. Secondly, the number of embryos transferred will also affect the pregnancy outcome of the freeze-thaw embryo transfer cycle. The more the number of embryos transferred or the more high-quality embryos transferred, the higher the pregnancy rate and LBR will be obtained. Finally, this study spent the most space to discuss the effect of embryo quality on pregnancy outcome during freeze-thaw embryo transfer cycle. It includes four elements: embryo cell number, embryo grade, embryo fragmentation and embryo uniformity. Better CPR, IR, and LBR can be obtained by transferring 7–9 cell embryos. Transfer of grade 1 embryos can achieve higher CPR, IR, and LBR. Both fragmentation and asymmetrical cleavage lead to a decrease in CPR, IR, and LBR.

## Data Availability

The raw data supporting the conclusions of this article will be made available by the authors, without undue reservation.
